# Risk factors for intensive care unit admission in patients with severe leptospirosis: a comparative study according to patients’ severity

**DOI:** 10.1186/s12879-016-1349-x

**Published:** 2016-02-01

**Authors:** Elizabeth De Francesco Daher, Douglas Sousa Soares, Anna Tereza Bezerra de Menezes Fernandes, Marília Maria Vasconcelos Girão, Pedro Randal Sidrim, Eanes Delgado Barros Pereira, Natalia Albuquerque Rocha, Geraldo Bezerra da Silva

**Affiliations:** 1 Departament of Internal Medicine, School of Medicine, Division of Nephrology, Federal University of Ceará, Rua Vicente Linhares, 1198, Fortaleza, Ceará CEP: 60135-270 Brazil; 2 Medical Sciences Graduate Program, School of Medicine, Federal University of Ceará, Rua Vicente Linhares, 1198, Fortaleza, Ceará CEP: 60135-270 Brazil; 3 Department of Medicine, University of Texas Southwestern Medical Center, Dallas, TX USA; 4 Public Health Graduate Program, School of Medicine, Health Sciences Center, University of Fortaleza, Fortaleza, Ceará Brazil

**Keywords:** Leptospirosis, Weil’s disease, Acute kidney injury, Intensive care unit, Mortality

## Abstract

**Background:**

The aim of this study is to investigate predictive factors for intensive care unit (ICU) admission among patients with severe leptospirosis.

**Methods:**

This is a retrospective study with all patients with severe leptospirosis admitted to a tertiary hospital. Patients were divided in ICU and ward groups. Demographical, clinical and laboratory data of the groups were compared as well as acute kidney injury (AKI) severity, according to the RIFLE criteria (R = Risk, I = Injury, F = Failure, L = Loss, E = End-stage kidney disease).

**Results:**

A total of 206 patients were included, 83 admitted to ICU and 123 to ward. Mean age was 36 ± 15.8 years, with 85.9 % males. Patients in ICU group were older (38.8 ± 15.7 vs. 34.16 ± 15.9 years, *p* = 0.037), had a shorter hospital stay (4.13 ± 3.1 vs. 9.5 ± 5.2 days, *p* = 0.0001), lower levels of hematocrit (29.6 ± 6.4 vs. 33.1 ± 8.6 %, *p* = 0.003), hemoglobin (10.2 ± 2.4 vs. 11.6 ± 1.9 g/dL, *p* < 0.0001), and platelets (94,427 ± 86,743 vs. 128,896 ± 137,017/mm^3^, *p* = 0.035), as well as higher levels of bilirubin (15.0 ± 12.2 vs. 8.6 ± 9.5 mg/dL, *p* = 0.001). ICU group also had a higher frequency of severe AKI (RIFLE-“Failure”: 73.2 % vs. 54.2 %, *p* < 0.0001) and a higher prevalence of dialysis requirement (57.3 % vs. 27.6 %, *p* < 0.0001). Mortality was higher among ICU patients (23.5 % vs. 5.7 %, *p* < 0.0001). Independent predictors for ICU admission were tachypnea (*p* = 0.027, OR = 13, CI = 1.3–132), hypotension (*p* = 0.009, OR = 5.27, CI = 1.5–18) and AKI (*p* = 0.029, OR = 14, CI = 1.3–150). Ceftriaxone use was a protective factor (*p* = 0.001, OR = 0.13, CI = 0.04–0.4).

**Conclusions:**

Independent risk factors for ICU admission in leptospirosis include tachypnea, hypotension and AKI. Ceftriaxone was a protective factor for ICU admission, suggesting that its use may prevent severe forms of the disease.

## Background

Leptospirosis is one of the most important zoonosis in the world [1]. The disease may manifest as a broad spectrum of signs and symptoms, with a sudden onset of headache, high-degree fever, malaise, myalgia, conjunctival suffusion and transient rash [1]. The severe form is characterized by jaundice, acute kidney injury (AKI) and hemorrhage, particularly in the lungs, known as Weil’s disease [1, 2]. It is mainly caused by serovars *Icterohaemorrhagiae*, *Copenhageni*, *Lai* and others [1, 2]. Mortality from severe leptospirosis is high, ranging from 5 to 20 %, even when optimal treatment is provided^3^. Leptospirosis-associated AKI has peculiar characteristics: it is usually non-oliguric and associated with hypokalemia or normokalemia [1, 3, 4].

Intensive Care Unit (ICU) admission is often necessary for patients with severe leptospirosis. In a recent Brazilian study, leptospirosis was among the most frequent infectious diseases that required ICU [5]. Mortality rates remain high, despite of treatments instituted [3, 6]. It was demonstrated that early dialysis reduces mortality and complications associated with leptospirosis in the ICU [7].

Early identification of severe or potentially severe cases of leptospirosis, and then possible ICU admission need, is of crucial importance to enhance more aggressive treatment and decrease mortality, which is still unacceptable high in this disease. As leptospirosis is endemic in many tropical and developing countries, a more adequate classification and approach to patients can probably impact on economic issues. A mortality decrease in leptospirosis, for example, is favorable to labor, once great part of leptospirosis patients are young man, and it is possible to avoid years of life lost.

Hence, the aim of this study was to investigate predictive factors for ICU admission among patients with severe leptospirosis.

## Methods

### Studied population

The study included all patients with confirmed diagnosis of severe leptospirosis (Weil’s disease) consecutively admitted to São José Infectious Diseases Hospital, in Northeast Brazil, from 2000 to 2013. *Study Design*

This is a cross-sectional study. Data on hospital admission were obtained from medical records. They were initially admitted to the emergency department, and then transferred to another unit (ward or ICU). We also grouped these patients according to the site of care: those transferred to the ICU and to ward. A comparison of demographical, clinical and laboratory data between the two groups was performed in order to investigate differences between them and factors associated with ICU admission.

### Case definition

Leptospirosis cases were defined as the presence of positive serology through microscopic agglutination test (MAT) higher than 1:800. All patients had also epidemiological and clinical history compatible with leptospirosis.

### Inclusion criteria

Inclusion criteria were: patients ≥ 18 years-old, positive serology for leptospirosis, admission at São José Infectious Diseases Hospital during the study period.

### Exclusion criteria

Exclusion criteria were: patients ≤ 18 years-old, negative serology for leptospirosis, incomplete data on medical records, referral to other hospitals and hospital stay less than 24 h.

### Studied parameters

Demographic characteristics such as age, gender, time between initial symptoms and hospital admission as well as length of hospital stay were recorded. The clinical investigation included a record of all clinical signs and symptoms presented by each patient on hospital admission and during hospital stay, medications used, dialysis requirement and co-infections.

Laboratory data on hospital admission included an assessment of serum urea, creatinine, sodium, potassium, total bilirubin, direct bilirubin, indirect bilirubin, aspartate aminotransferase (AST), alanine aminotransferase (ALT), lactate dehydrogenase (LDH), hemoglobin, hematocrit, leukocyte count, platelets count and arterial blood gas analysis.

### Definitions

Acute kidney injury (AKI) was defined according to RIFLE criteria (R = Risk, I = Injury, F = Failure, L = Loss, E = End-stage kidney disease) [8]. Thrombocytopenia was defined as platelets count lower than 150,000/mm^3^ and anemia as hemoglobin <12 g/dL. The occurrence of metabolic acidosis was considered when pH < 7.35 and HCO_3_ < 20 mEq/L, and severe metabolic acidosis when pH < 7.10. Tachypnea was defined as a respiratory rate higher than 25 per minute. Oliguria was defined as urine output < 400 ml/day after 24 h of effective hydration. Hypotension was defined as mean arterial blood pressure (MAP) < 60 mmHg, and therapy with vasoactive drugs was initiated when MAP remained lower than 60 mmHg despite the use of endovenous fluids. Hypertension was defined as systolic pressure ≥130 mmHg and/or diastolic pressure ≥85 mmHg.

### Statistical analysis

The results were expressed through tables, mean ± standard deviation (SD). All data were analyzed with the program SPSS version 20.0 (Chicago, IL, USA). Comparison between the two groups was performed using Pearson’s Chi-square test and Student’s *T* test. Mann–Whitney test was used for parameters with a non-normal distribution. A logistic regression model was used for quantitative variables. Adjusted odds ratios (OR) and 95 % confidence intervals (CI) were calculated. A multivariate logistic regression was performed to analyze the possible risk factors associated with ICU admission. The parameters included in the multivariate model were those that showed a significance level in the univariate analysis. Significance level was set at 5 % (*p* values ≤ 0.05).

### Ethics

The protocol of this study was approved by the Ethics Committee of São José Infectious Diseases Hospital, Fortaleza, Ceará, Brazil.

## Results

### Patients’ characteristics

Among 206 patients included in the study, eighty-three were included in the ICU group and 123 in the ward group. There was a predominance of males (85.9 %), and mean age was 36 ± 15.8 years. Complete evaluation of demographic data is presented in Table 1.

**Table 1:** Comparison of demographic data between patients with severe leptospirosis admitted to ICU and ward

	ICU (*N* = 83)	Ward (*N* = 123)	*p*
Age (years)	38.8 ± 15.7	34.1 ± 15.9	0.037
Gender			
Male	72 (86.7 %)	105 (85.4 %)	0.840
Female	11 (13.3 %)	18 (14.6 %)	
Hospital stay (days)	4.13 ± 3.1	9.5 ± 5.2	0.0001
Time between onset of symptoms and hospitalization (days)	7 ± 4	7.22 ± 4.11	0.224
Mortality (%)	23.5	5.7	<0.0001

Pearson’s Chi-square test, T-Student test and Mann–Whitney test were used to perform this comparison. *P* values ≤ 0.05 were considered statistically significant

### Signs and symptoms at admission

Main signs and symptoms at hospital admission were fever (87.1 %), myalgia (77.2 %), jaundice (71.7 %), headache (65.5 %), calf pain (48.5 %), asthenia (45.0 %) and diarrhea (41.5 %). Higher levels of heart and respiratory rates were observed in ICU group (105.0 ± 19.5 vs. 94.7 ± 19.0/min, *p* = 0.008; 31.8 ± 13.0 vs. 25.0 ± 8.8/min, *p* = 0.004, respectively), as well as lower levels of systolic blood pressure (106.3 ± 27 vs. 115.2 ± 18 mmHg, *p* = 0.018), as summarized in Table 2.

**Table 2:** Comparison of signs, symptoms and vital signs among patients with severe leptospirosis admitted to ICU and ward

	ICU (*N* = 48)	Ward (*N* = 123)	*p*
Signs and symptoms			
Choluria	7 (16.6 %)	54 (44.7 %)	0.001
Crackles	1 (2.1 %)	15 (13 %)	0.043
Diarrhea	12 (25 %)	52 (42.3 %)	0.855
Dyspnea	18 (37.5 %)	33 (27.6 %)	0.287
Fever	33 (68.8 %)	115 (94.3 %)	<0.0001
Hematemesis	7 (14.6 %)	7 (5.7 %)	0.068
Hematuria	3 (8.3 %)	8 (7.3 %)	0.759
Hemoptysis	8 (16.7 %)	9 (8.1 %)	0.625
Jaundice	30 (64.5 %)	87 (71.5 %)	0.998
Myalgia	27 (58.3 %)	104 (84.6 %)	<0.0001
Oliguria	10 (22.9 %)	32 (26.8 %)	0.699
Conjunctival suffusion	4 (10.4 %)	22 (17.9 %)	0.305
Tachypnea	15 (33.3 %)	15 (13 %)	0.004
Vital signs			
SBP (mmHg)	106.28 ± 27	115.17 ± 18	0.018
DBP (mmHg)	64.53 ± 18.7	71.3 ± 14.8	0.18
HR (/min)	105.03 ± 19.5	94.7 ± 19.05	0.008
RR (/min)	31.8 ± 13	25 ± 8.78	0.004

*SBP* systolic blood pressure, *DBP* diastolic blood pressure, *HR* Heart Rate, *RR* Respiratory Rate. Pearson’s Chi-squared test, T-Student test and Mann–Whitney test were used. *P* values ≤ 0.05 were considered statistically significant

### Comparison between ICU and ward group

Patients in the ICU group were older than ward group (38.8 ± 15.7 vs. 34.2 ± 15.9 years, *p* = 0.037). Average length of hospital stay was shorter in the ICU group (4.13 ± 3.1 vs. 9.5 ± 5.2 days, *p* = 0.001). Patients admitted to ICU also presented lower levels of hematocrit (29.6 ± 6.4 vs. 33.1 ± 8.6 %, *p* = 0.003), hemoglobin (10.2 ± 2.4 vs. 11.6 ± 1.9 g/dL, *p* < 0.0001), platelets (94,427 ± 86,743 vs. 128,896 ± 137,017/mm^3^, *p* = 0.035), as well as higher levels of total bilirubin, direct and indirect bilirubin (15.0 ± 12.2 vs. 8.6 ± 9.5 mg/dL, *p* = 0.001; 9.2 ± 8.5 vs. 5.9 ± 7.1 mg/dL, *p* = 0.018; 4.5 ± 6.1 vs. 2.4 ± 3.2 mg/dL, *p* = 0.013, respectively) on hospital admission, as summarized in Table 3. ICU patients also presented higher prevalence of hypertension (68.0 vs. 43.9 %, *p* = 0.006), hypotension (66.0 vs. 33.3 %, *p* < 0.0001), metabolic acidosis (60.5 vs. 36.5 %, *p* = 0.011) and severe metabolic acidosis (11.0 vs. 0 %, *p* = 0.02) on hospital admission.

**Table 3:** Comparison of laboratory data between patients with severe leptospirosis admitted to ICU and wards

Parameters	ICU	Non-ICU	*p*
	*N* = 83	*N* = 123	
Creatinine (mg/dl)	3.97 ± 2.25	2.97 ± 2.4	0.004
Urea (mg/dl)	131.8 ± 68	101.2 ± 72.5	0.004
Potassium (mEq/L)	3.91 ± 0.85	3.9 ± 1.07	0.951
Sodium (mEq/L)	133 ± 6.61	130 ± 19	0.317
AST (UI/L)	169.8 ± 199	132.13 ± 183	0.285
ALT (UI/L)	101.83 ± 90	101.90 ± 153	0.998
LDH (UI/L)	810.25 ± 539	671.94 ± 420	0.416
Total bilirubin (mg/dl)	15 ± 12.2	8.6 ± 9.5	0.001
Direct bilirubin (mg/dl)	9.2 ± 8.5	5.9 ± 7.11	0.018
Indirect bilirubin (mg/dl)	4.48 ± 6.08	2.38 ± 3.22	0.013
Hematocrit (%)	29.6 ± 6.4	33.1 ± 8.63	0.003
Hemoglobin (g/dl)	10.2 ± 2.4	11.6 ± 1.95	<0.001
WBC (10^3^/mm^3^)	14.30 ± 7.70	14.95 ± 1.82	0.772
Platelets (10^3^/mm^3^)	94.42 ± 86.74	128.89 ± 137.01	0.035
pO_2_ (mmHg)	87.3 ± 32	83 ± 34	0.542
pCO_2_ (mmHg)	31.81 ± 9.4	32.64 ± 8.5	0.636
pH	7.35 ± 0.08	7.36 ± 0.07	0.127
Bicarbonate (mEq/L)	17.89 ± 4.82	18.8 ± 4.89	0.330
SaO_2_ (%)	93.82 ± 5.7	91.6 ± 10.17	0.190

*AST* aspartate aminotransferase, *ALT* alanine aminotransferase, *LDH* lactate dehydrogenase, *WBC* white blood cells, *PO*_*2*_ O_2_ partial pressure, *PCO*_*2*_ PCO_2_ partial pressure, *SaO*_*2*_ O_2_ saturation

Chi-squared test, T-Student test and Mann–Whitney test were used to perform this comparison. Values expressed as mean ± SD and percentage. *P* values ≤ 0.05 were considered statistically significant

ICU group had higher levels of serum creatinine (3.97 ± 2.25 vs. 2.97 ± 2.4 mg/dL, *p* = 0.004) and serum urea (131.8 ± 68 vs. 101.2 ± 72.5 mg/dL, *p* = 0.004) on hospital admission. Oliguria was not significantly different between ICU group and ward group (22.9 % v. 26.8 %, *p* = 0.699, respectively). The ICU group also presented higher prevalence of AKI (93.9 % vs. 69.9 %, *p* < 0.0001), with a higher number of severe forms according to RIFLE criteria (“Risk” 4.9 % vs. 7.5 %, “Injury” 17.1 % vs. 10.0 % e “Failure” 73.2 % vs. 54.2 %, *p* < 0.0001). Need of dialysis was more frequent in this group (57.3 % vs. 27.6 %, *p* < 0.0001) when compared to ward group, as presented in Table 4.

**Table 4:** Comparison of acute kidney injury and dialysis requirement between patients with severe leptospirosis admitted to ICU and wards

	ICU (*N* = 83)	Ward (*N* = 123)	*p*
AKI	77 (93.9 %)	85 (69.9 %)	<0.0001
Dialysis	47 (57.3 %)	33 (27.6 %)	<0.0001
RIFLE			
Risk	3 (4.9 %)	9 (7.5 %)	
Injury	14 (17.1 %)	12 (10 %)	<0.0001
Failure	60 (73.2 %)	66 (54.2 %)	

Chi-square test was performed to make this comparison. *P* values ≤ 0.05 were considered statistically significant

### Treatment

Need of vasoconstrictors and use of diuretics were higher in ICU group (57.8 vs. 12.3 %, *p* < 0.0001; 49.4 vs. 26.1 %, *p* = 0.006, respectively). Ceftriaxone use was significantly higher in the ward group (67.6 vs. 41 %, *p* = 0.009), while the use of crystalline penicillin was similar in both groups, as summarized in Table 5.

**Table 5:** Comparison of treatments instituted for patients with severe leptospirosis admitted to ICU and ward

	ICU (*N* = 83)	Ward (*N* = 123)	*p*
Hemoderivatives	44 (54 %)	58 (47.7 %)	0.052
Vasoconstrictors	47 (57.8 %)	15 (12.3 %)	<0.0001
Ceftriaxone	34 (41 %)	83 (67.6 %)	0.009
Penicillin	48 (59 %)	60 (49.1 %)	0.354
Diuretics	41 (49.4 %)	32 (26.1 %)	0.006

Pearson’s Chi-squared test was used to make the comparison. P values ≤ 0.05 were considered statistically significant

### Risk factors for ICU admission

In multivariate analysis, independent predictors for ICU admission were: tachypnea (*p* = 0.027, OR: 13, CI = 1.3 - 132), hypotension (*p* = 0.009, OR: 5.27, CI = 1.5 - 18) and AKI severity (*p* = 0.029, OR: 14, CI = 1.3 - 150). On the other hand, ceftriaxone use was a protective factor (*p* = 0.001, OR = 0.13, CI = 0.04 – 0.4) for ICU admission, as described in Table 6.

**Table 6:** Independent risk factors for ICU admission among patients with severe leptospirosis (multivariate analysis)

Multivariate analysis	*p*	OR/ 95 % CI
Tachypnea	0.027	13 (1.3 – 132)
Hypotension	0.009	5.27 (1.5 – 18)
AKI	0.029	14 (1.3 – 150)
Ceftriaxone	0.001	0.13 (0.04-0.4)

*OR* odds ratio. 95 % CI: 95 % confidence interval. Logistic regression and multivariate analysis were performed to make this comparison. *P* values ≤ 0.05 were considered statistically significant

### Mortality

Overall mortality was 12.7 %, and it was higher in ICU group (23.5 vs. 5.7 %, *p* < 0.0001).

## Discussion

Predictors for ICU admission among patients with severe leptospirosis include tachypnea, hypotension and severe AKI, which can be identified at hospital admission and then subside requirement for a more aggressive treatment. The use of ceftriaxone was a protective factor against ICU admission, which suggests an important effect on decreasing disease severity.

Leptospirosis is a public health problem, with higher prevalence in tropical areas, including Brazil [4, 9]. The disease can present with severe manifestations and high mortality. Therefore it is important to characterize predictors of intensive care need in order to early identify potentially fatal cases and to provide a more careful approach. This is the first study in our region to investigate the differences between patients with leptospirosis admitted to a ward service and ICU.

In the present study patients admitted to the ICU were older, which may reflect an association between advanced age and disease severity, as previously reported [10–12]. In a recent study from India, older age was a predictor of mortality in leptospirosis [3]. Leptospirosis in elderly patients was associated with severe course and increased risk of mortality, mostly due to the high incidence of comorbidities in this population [12].

The length of hospital stay was shorter in ICU patients, and this is probably due to a high mortality in cases that demand intensive care. We observed that more critically ill patients usually died during the first days of ICU admission, despite early treatment. Previous studies have shown that early daily dialysis in patients with leptospirosis-associated AKI reduces mortality and, therefore, it would probably be beneficial in critically ill patients [7, 13].

In the present study, ICU patients presented higher levels of total, direct and indirect bilirubin than ward patients. In a French study with leptospirosis patients, bilirubinemia was higher in severe cases and jaundice was demonstrated to be a predictive factor of severity in leptospirosis according to multivariate analysis (*p* = 0.005, OR = 10.1, CI = 1.79 – 56.8) [14]. In this same study, dyspnea (respiratory rate > 24/min) was higher in severe cases (*p* < 0.0001). These findings confirm the data in our study and reinforce the role of tachypnea as independent predictor for ICU admission, according to multivariate analysis.

We have found higher prevalence of anemia and thrombocytopenia as well as higher levels of urea and creatinine in the ICU group. Thrombocytopenia is frequent in leptospirosis and contributes to hemorrhagic phenomena [2, 15]. Although rare, pancytopenia can also be found in leptospirosis’ patients [16], but when it occurs other diagnosis must be investigated, such as hematologic disorders and visceral leishmaniasis, especially in tropical countries. In a recent study conducted in our region with 374 leptospirosis patients, thrombocytopenia was found in 53.5 % of patients on admission and it was associated with length of disease and AKI, but it was not associated with higher mortality [15].

Oliguria is a frequent feature in patients with severe AKI and it is associated with more frequent pulmonary involvement and higher mortality in leptospirosis [2, 13, 17–20]. However, in the present study, oliguria was not associated with ICU admission, since there was no significant difference in frequency of oliguria between the two groups. Several hemodynamic changes that occur in leptospirosis, such as systemic vasodilation, renal vasoconstriction and elevation of aldosterone, associated with dehydration secondary to fever, vomiting and diarrhea could explain the onset of oliguria in leptospirosis patients [4, 21].

Hypotension was found to be a predictor of ICU admission in our study, according to multivariate analysis. Hemodynamic changes, such as increased vascular permeability secondary to cytokines and vasoactive mediators might also explain the onset of hypotension in severe cases. Low mean arterial pressure is well described as an important factor for the onset of AKI and disease severity in leptospirosis patients [22].

It is known that AKI severity in leptospirosis is associated with higher mortality [17, 23]. RIFLE and AKIN are good markers for the purpose of stratifying patients’ severity in leptospirosis. Worse AKI stages (RIFLE-F and AKIN-3) are associated with higher mortality [17]. In the present study, AKI RIFLE “Failure” was significantly higher in the ICU group.

The most recognized factors correlating with severity in leptospirosis are AKI, pulmonary involvement and electrolyte imbalances [24]. In the present study, independent risk factors associated with ICU admission were hypotension, tachypnea and AKI severity. In a recent study with 176 leptospirosis patients, independent predictors of severity were current cigarette smoking (OR  =  2.94 [CI 1.45-5.96]), delay >2 days between the onset of symptoms and the initiation of antibiotic therapy (OR  =  2.78 [CI 1.31-5.91]) and *Leptospira interrogans* serogroup *Icterohaemorrhagiae* as the infecting strain (OR  =  2.79 [CI 1.26-6.18]) [25]. Another study, conducted with 101 leptospirosis patients in India, showed that older age, delayed antibiotic therapy, higher bilirubin, aspartate aminotransferase, alkaline phosphatase, leucocyte count and aspartate/alanine aminotransferase ratio (AAR) were univariate predictors of mortality.

Ceftriaxone use was more frequent among patients admitted to ward and it was shown to be a protective factor for ICU admission, different from penicillin use, which was not different between groups. The use of antibiotics in leptospirosis has been widely debated. Numerous studies have been conducted to investigate the role of antibiotics in leptospirosis, with controversial results, and the current consensus is that antibiotics must be used, since it probably brings more benefits than harm, reducing the time of hospital stay, the duration of fever and slowing disease symptoms in general [26–30]. It is possible that antibiotics have an important role in immune response modulation in leptospirosis, which could partially explain the observation of a better evolution in patients treated with penicillin [31–33]. Nonetheless, mortality does not seem to be significantly influenced by antibiotic use [34].

As expected, in the present study, mortality was higher among ICU patients (23.5 % vs. 5.7 %, *p* < 0.0001) when compared to ward group. Some studies performed in our region have found mortality rates varying from 12 to 20 % in leptospirosis, which were lower than those found in the present study [35]. Even higher mortality rates have been described in some cases. In a study performed with 60 leptospirosis ICU patients between 2002 and 2003, mortality was 52 %, which was remarkably higher than average mortality in that ICU in the period [36]. These data reinforce the importance of determining predictors of ICU admission in leptospirosis.

## Conclusion

Tachypnea, hypotension and AKI are risk factors for ICU admission, and ceftriaxone use is a protective factor. These results alert to early identification of the above mentioned factors and then a more aggressive treatment, including early antibiotics use and renal replacement therapy when needed to possibly decrease mortality.

### Study limitations

Main limitations of this study derive from its retrospective nature. Some data from patient’s records were not available on admission. Urine output and urinalysis were particularly poorly available in the ward group, since non-critical care units frequently do not accurately measure and analyze urine output. Further prospective studies are then necessary to continue the investigation of leptospirosis predictors of ICU admission.

## Abbreviations

AKI: Acute kidney injury
ALT: Alanine aminotransferase
AST: Aspartate aminotransferase
ICU: Intensive care unit
LDH: Lactate dehydrogenase
OI: Odds ratio
MAP: Mean arterial blood pressure
MAT: Microscopic agglutination test
OR: Odds ratio
RIFLE: Acrony for Risk, Injury, Failure, Loss, End-stage renal disease
SPSS: Statistic package for social science
